# Metabolic plasticity of cancer stem cells

**DOI:** 10.18632/oncotarget.6177

**Published:** 2015-10-19

**Authors:** Ming Luo, Max S. Wicha

**Affiliations:** University of Michigan Comprehensive Cancer Center and Department of Internal Medicine, University of Michigan, Ann Arbor, Michigan, USA

**Keywords:** cancer stem cells, glycolysis, oxidative metabolism

Cancer stem cells (CSCs) are a small population of tumor cells that drive tumor growth, metastasis and treatment resistance. In the early 1900s, Dr. Otto Warburg made the seminal discovery that tumor cells, in contrast to their normal counterparts, consume large amounts of glucose with resultant lactate production, even in the presence of oxygen. Such increased aerobic glycolysis in tumor cells is well known (the so-called “Warburg effect”). Despite increased aerobic glycolysis in the tumor bulk, the metabolic phenotype of cancer stem cells is less clear. Recent studies in breast cancer have described breast CSCs (BCSCs) as more glycolytic compared to corresponding bulk tumor cells [1, 2]. However, studies in other tumor types, including pancreatic cancer and leukemia, demonstrated that quiescent or slow-cycling tumor initiating cells are dependent on mitochondrial respiration for tumor development and maintenance [3, 4].

Although Warburg proposed that mitochondrial respiration in cancer cells is irreversibly damaged, a growing body of evidence challenges this view. A recent study from Lisanti and colleagues present further evidence that mitochondrial mass can serve as a metabolic biomarker of BCSCs [5]. In this study, the authors utilized MCF7 cells that overexpress WNT1 and FGF3, two well-known signaling proteins that promote EMT and stem-like properties. Not surprisingly, they found that enhanced WNT/FGF signaling promoted stem-like properties including tumorsphere formation. Interestingly, proteomic analysis of the MCF7 cells, with or without WNT1/FGF3 overexpression, revealed that mitochondria-related proteins and enzymes involved in the TCA cycle were up-regulated by WNT1/FGF3 in MCF7 cells. This observation suggested a link between breast CSCs and mitochondrial respiration. Indeed, following MitoTracker Deep-Red labeling, the cells with the highest MitoTracker signal (high mitochondria mass) displayed the greatest capacity for the formation of tumorspheres, a CSC property. Thus, breast cancer cells with stem-like properties may rely on mitochondrial respiration for their energy requirements.

An intriguing finding from the above study is that a wide variety of enzymes involving glycolysis and the pentose phosphate pathway (PPP) were also up-regulated in MCF7 cells upon WNT1/FGF3 overexpression. Although the functional significance of these increased glycolytic enzymes was not addressed in the paper, it is likely that BCSCs may be endowed with capacity for both aerobic glycolysis and oxidative phosphorylation (OXPHOS). However, the metabolic phenotype of CSCs in each type of tumor may be regulated by the tumor microenvironment. In support of this, a recent study by Dupuy et al. demonstrated that murine cancer cells (4T1) with the highest metastatic potential displayed enhanced capacity for both glycolytic and oxidative metabolism. In contrast, tumor cells of the same genetic background that lack metastatic capacity, (67NR), exhibited a much lower capacity for both glycolytic and oxidative metabolism [6]. Importantly, this study also demonstrated that 4T1 tumor cells display extensive metabolic heterogeneity, engaging glycolysis or oxidative phosphorylation depending on their site of metastasis. This study suggests that BCSCs, which are the cells that mediate tumor metastasis, may similarly be endowed with capacity for metabolic plasticity.

It has recently been shown that BCSC exist in two interchangeable states: epithelial-like (MET) and mesenchymal-like (EMT). The MET BCSCs are characterized by their high aldehyde dehydrogenase (ALDH) activity and proliferative capacity while EMT BCSCs are characterized by (CD24-CD44^hi^) cell surface marker expression and by their quiescent, slow-cycling state [7]. The transition between EMT and MET like CSC states is regulated by the tumor microenvironment, and the plasticity to transition between these states may play an important role in tumor metastasis. Although it remains to be determined if EMT and MET BCSCs display metabolic differences under different environmental conditions, the property of metabolic plasticity observed in these cells adds another layer of complexity to our understanding of CSC biology.

**Figure 1:**
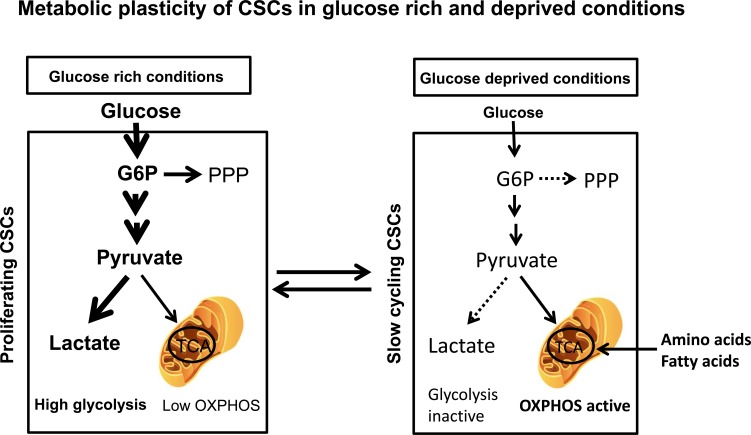
Metabolic plasticity of aerobic glycolysis and mitochondrial oxidative metabolism in CSCs under differing environmental conditions In glucoserich environments, proliferating CSCs primarily utilize aerobic glycolysis for their bioenergetics needs, while in glucose (and oxygen) deprived conditions, CSCs shift to a quiescent, slowcycling state relying on mitochondrial oxidative metabolism for their energy needs.

Based on recent published literature, we present a model illustrating CSC metabolic plasticity and its regulation by the tumor microenvironment (Figure 1). In glucose and oxygen rich conditions, CSCs primarily rely on aerobic glycolysis as originally proposed by Warburg. This increased glycolysis also helps offset the oxidative stress generated in proliferating tumor cells including CSCs. However, in tumor microenvironments that restrict proliferation such as glucose deprivation and hypoxia, CSCs shift to a metabolic state capable of supporting quiescent, slow-cycling CSCs, relying heavily on mitochondrial oxidative metabolism to support their energy needs. The capacity of CSCs to alter their metabolic profile in response to the tumor microenvironment may allow them to survive in the hostile environments that they encounter at metastatic sites. If this is the case then future therapies designed to attack CSCs may need to simultaneously target tumor glycolytic and mitochondrial oxidative metabolism.
